# Co-Deformation Process of Cu and Fe Phases in Cu-10Fe Alloy During Cold Rolling

**DOI:** 10.3390/ma18112547

**Published:** 2025-05-28

**Authors:** Wei Chen, Xiaona Hu, Jiawei Wang, Qiuxiang Liu, Dan Wu, Jiang Jiang, Qiang Hu, Deping Lu, Jin Zou

**Affiliations:** 1 Institute of Applied Physics, Jiangxi Academy of Sciences, 7777 Changdong Avenue, Nanchang 330096, China; chenw@alum.imr.ac.cn (W.C.); xnhu13s@163.com (X.H.); jww@whu.edu.cn (J.W.); qx0813@126.com (Q.L.); bird5810761@163.com (D.W.); superjj1981@163.com (J.J.); huqiang1225@163.com (Q.H.); ludeping61@163.com (D.L.); 2 Jiangxi Key Laboratory of Advanced Copper-Based Materials, 7777 Changdong Avenue, Nanchang 330096, China

**Keywords:** Cu-Fe alloy, deformation mechanism, morphological evolution, texture development, EBSD

## Abstract

Cu-Fe in situ composites often face challenges in achieving high strength during cold rolling due to the inefficient transformation of partial Fe phases into fibrous structures. To uncover the underlying mechanisms, this study systematically investigates the co-deformation behavior of Cu and Fe phases in a Cu-10Fe alloy subjected to cold rolling at various strains. Through microstructure characterization, texture analysis, and mechanical property evaluation, we reveal that the Cu matrix initially accommodates most applied strain (*ε*_vm_ < 1.0), forming shear bands, while Fe phases (dendrites and spherical particles) exhibit negligible deformation. At intermediate strains (1.0 < *ε*_vm_ < 4.0), Fe phases begin to deform: dendrites elongate along the rolling direction, and spherical particles evolve into tadpole-like morphologies under localized shear. Concurrently, dynamic recrystallization occurs near Fe phases in the Cu matrix, generating ultrafine grains. Under high strains (*ε*_vm_ > 4.0), Fe dendrites progressively transform into filaments, whereas spherical Fe particles develop long-tailed tadpole-like structures. Texture evolution indicates that Cu develops a typical copper-type rolling texture, while Fe forms α/γ-fiber textures, albeit with sluggish texture development in Fe. The low efficiency of Fe fiber formation is attributed to the insufficient strength of the Cu matrix and the elongation resistance of spherical Fe particles. To optimize rolled Cu-Fe in situ composites, we propose strengthening the Cu matrix (via alloying/precipitation) and suppressing spherical Fe phases through solidification control. This work provides critical insights into enhancing Fe fiber formation in rolled Cu-Fe systems for high-performance applications.

## 1. Introduction

Cu-based alloys containing bcc metal fibers (Cu-Cr, Cu-Fe, Cu-Nb, …) prepared by heavy mechanical working are important industrial materials [1,2,3]. Such alloys possess a high strength contributed by a combination of work hardening and fiber strengthening [4,5,6], and retain a high conductivity due to the limited solubility of these bcc elements in the Cu matrix [7,8,9,10]. Among alloys of this type, the Cu-Fe systems are of particular interest because of the low cost of iron. Over the past three decades, a variety of mechanical working methods have been applied to prepare Cu-Fe alloys with fibrous microstructures, such as drawing [11], rolling [12], high-pressure torsion [13,14] and equal-channel angular pressing [15], and the microstructure and mechanical or physical properties of the samples have been thoroughly investigated [16,17,18,19,20,21,22,23,24,25].

Many studies have focused on the drawing of Cu-Fe alloys because it is the most widely used wire-forming technology. It is shown that the Fe phase can transform into aligned filaments with a ribbon-like cross section as the drawing strain becomes sufficiently large [5,11]. The strength of these wires increases with decreasing Fe filament spacing, leading to a Hall–Petch type relation [26,27,28]. According to the literature [5,16], tensile strengths as high as 1 GPa or more can be achieved in the Cu-Fe alloys after high drawing strains. Rolling is the most commonly used sheet-forming technology, but it is much less studied than drawing in Cu-Fe alloys. Several studies [29,30] show that the tensile strength of Cu-Fe alloy sheets prepared by rolling is lower than that of Cu-Fe alloy wires prepared by drawing at the same applied strains. The lower tensile strength of the Cu-Fe alloy sheets is attributed to the presence of coarser Fe filaments [29,30] or other Fe phases with peculiar morphology, such as lens-like or tadpole-like Fe particles [24]. While refining Fe filaments can enhance strength via Hall–Petch mechanisms [26,27,28], the persistence of spherical or tadpole-like Fe particles disrupts stress transfer and limits strength improvements. Co-existing refined filaments and spherical particles create heterogeneous strain localization, reducing overall ductility and strength [30]. Recent work by Hong et al. [24] reveals that lens-shaped Fe particles in rolled alloys contribute minimally to strengthening. The finite element simulation of the Cu-Fe alloy extrusion process [27] and the deformation behavior of Cu-Fe-P alloy under low-temperature conditions [28] highlight the evolution of the material’s complex microstructure, encompassing deformed and recrystallized grains, strain localization, and heterogeneous deformation. However, the fundamental mechanisms governing the co-deformation of Cu matrix and Fe phases (dendrites vs. spherical particles) during rolling remain poorly understood, particularly regarding strain partitioning, texture evolution, and recrystallization effects.

The objective of this study is to systematically investigate the co-deformation mechanisms of Cu and Fe phases in cold-rolled Cu-10Fe alloys, focusing on the morphological evolution, texture development, and mechanical behavior. The novelty lies in (i) identifying three distinct co-deformation stages correlated to Fe morphology evolution, (ii) characterizing the texture evolution of the Cu and Fe phases during cold rolling, and (iii) revealing the relationships between Fe phase geometry and fiber formation efficiency. These findings enable targeted strategies for microstructural optimization not previously achievable.

## 2. Experimental Procedures

The experimental Cu-10Fe (wt.%) alloy was prepared from electrolytic copper (99.99 wt.%) and industrial pure iron (99.97 wt.%). After melting the raw materials in a magnesia crucible in the vacuum medium-frequency induction furnace, the alloy melt was cast into a graphite mold with an inner diameter of 60 mm and a length of 120 mm. The overheating temperature during melting was approximately 1250–1350 °C, which ensured complete dissolution of Fe into the Cu melt while avoiding excessive evaporation or oxidation. The casting temperature was maintained at 1150–1200 °C to ensure proper fluidity of the melt for filling the graphite mold. Specimens with dimensions of 10 mm in thickness, 120 mm in casting direction and 30 mm in width were cut from the center of the cast ingot. These specimens were cold rolled for several passes, and each pass reduction in thickness was 20~50%. The rolling direction (RD) and normal direction (ND) always correspond to the casting direction and thickness direction, respectively. The applied rolling strain is given by the Von Mises equivalent $\varepsilon_{\mathrm{VM}}=\left|2/\sqrt{3}\ln\left(1+r\right)\right|$, where *r* is the rolling reduction. The microstructure and properties of the samples were examined at strains of *ε*_vm_ = 0, 1, 2, 3, 4 and 5, respectively. A schematic diagram illustrating the key steps of casting, cold rolling, and specimen extraction can be found in the Supplementary Material (Figure S1).

The Vickers hardness (HV) measurements were carried out on a Future Tech FM-700 microhardness tester (Future-Tech Corp., Kanagawa, Japan) under the load of 50 g with a holding time of 10 s. The electrical conductivity was measured by a Shanghai jingmi ZY9987 digital micro-ohmmeter (Shanghai Precision & Scientific Instrument Co., Ltd., Shanghai, China), and the samples used for this test, with dimensions of 100 mm (length) × 5 mm (width) × 0.13 mm (thickness), were cut from the center of the as-cast plates and cold rolled sheets by wire-electrode cutting. Uniaxial tensile tests were performed on an Instron 5982 testing machine (Instron, Norwood, MA, USA) at a strain rate of 1 × 10^−3^ s^−1^. The tensile samples have a gauge length of 100 mm, a width of 20 mm and a thickness of 0.13 mm. Vickers hardness and electrical conductivity measurements were repeated five times per sample, and average values are reported. Tensile tests were conducted on three specimens per strain condition to ensure statistical reliability.

The microstructure of the samples was examined in the ND-RD cross section by a Nikon E600POL optical microscope (OM) (Nikon Corporation, Tokyo, Japan) and a Quanta FEG field emission scanning electron microscopy (SEM) (Thermo Fisher Scientific, Hillsboro, OR, USA). Samples for microstructure observation were chemically etched using a solution of 4 mL HNO_3_ and 96 mL C_2_H_5_OH for 15–20 s. Orientation imaging microscopy in Cu and Fe phases was obtained by electron back-scatter diffraction (EBSD). EBSD measurements were carried out using SEM (Quanta FEG), where a 20-kV electron beam was scanned with a step of 0.2 μm (unless otherwise noted) on the polished material surface perpendicular to the transverse direction (TD). To characterize the bulk crystallographic texture, a large-area EBSD method was used, which was performed with step sizes of 5 μm on the TD-RD plane at the center of the specimen with an area of 25 mm^2^. It should be noted that the X-ray diffraction (XRD) method was not used in this study to characterize the bulk crystallographic texture, although it usually provides more reliable data compared to the EBSD method, especially when the microstructure presents large grains. The reason lies in that the XRD method has three problems in the texture studies of two phase alloys [31]: (1) It is difficult to correctly determine pole figures of the Fe phase with only 10% volume fraction, since the reflection strength depends on the volume of the considered phase. (2) The reflection peaks of the two phases may be close to each other, leading to problems of separating the peaks during pole figure calculation. (3) Since the Fe grains are strongly elongated along the rolling direction under high rolling strains, the anisotropic absorption of X-rays in the two phases may lead to errors in the calculated pole figures. The strains *ε*_vm_ = 0, 1, 2, 3, 4, 5 were investigated experimentally, but representative strains were selected for figures to avoid redundancy and highlight critical transitions (e.g., *ε*_vm_ = 0, 1, 3, 5). However, to fully address the microstructural evolution, we have included micrographs and EBSD data for *ε*_vm_ = 2 and 4 in the Supplementary Materials (Figures S2–S4).

## 3. Results

### 3.1. Morphological Evolution of Cu and Fe Phases

**Figure 1:**
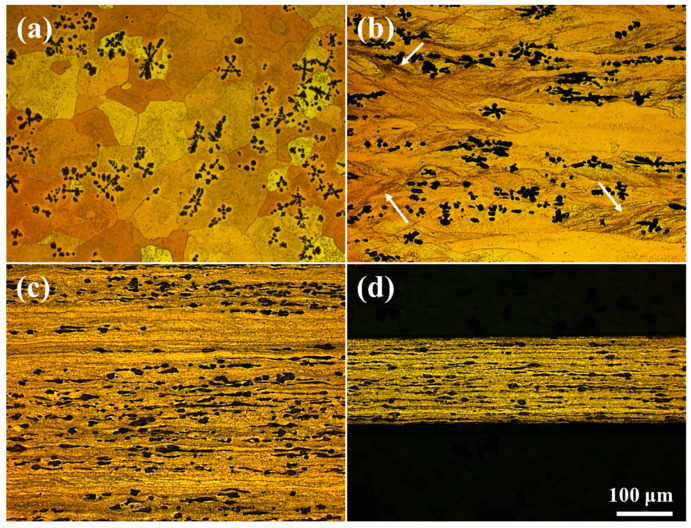
Optical micrographs of the Cu-10Fe alloy with different cold rolling strains. (**a**) *ε*_VM_ = 0, (**b**) *ε*_VM_ = 1.0, (**c**) *ε*_VM_ = 3.0, and (**d**) *ε*_VM_ = 5.0.

Figure 1 shows the morphological evolution of the Cu phase studied by OM observations. The Cu matrix in the as-cast state consists of almost equiaxed Cu grains with an average size of about 80 µm (Figure 1a). After applying a rolling strain of 1.0 (Figure 1b), the equiaxed Cu grains elongate along the rolling direction, and a large number of shear bands (indicated by the white arrows) form within some of the Cu grains that contain a high density of Fe phase. The appearance of shear bands is an important feature of inhomogeneous deformation. As the rolling strain increases from 1.0 to 3.0 (Figure 1b,c), the elongated Cu grains transform into a lamellar structure, whose thickness (in ND) decreases from about 19.8 to 3.5 μm, at which point the shear band microstructure is barely observed. The Cu lamellae seem to become thinner when the rolling strain is increased to 5.0 (Figure 1d); however, more detailed microstructures in the Cu lamellae could not be detected from the OM observations.

**Figure 2:**
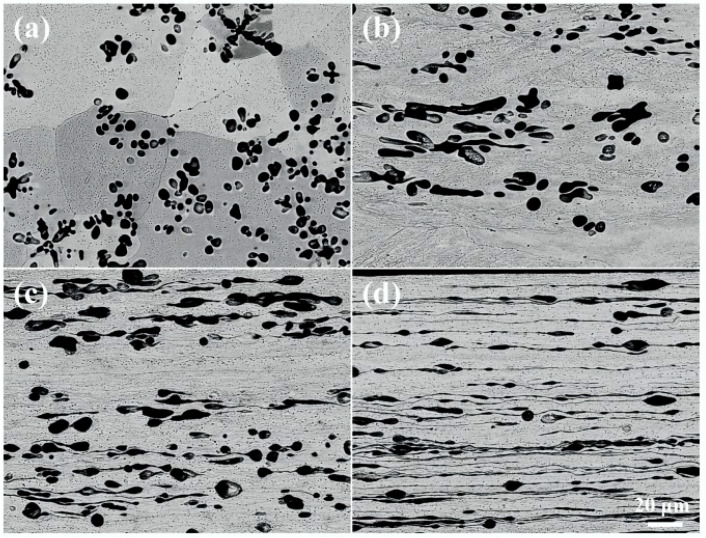
SEM images of the Cu-10Fe alloy with different cold rolling strains. (**a**) *ε*_VM_ = 0, (**b**) *ε*_VM_ = 1.0, (**c**) *ε*_VM_ = 3.0, and (**d**) *ε*_VM_ = 5.0.

Figure 2 illustrates the morphological evolution of the Fe phase observed by SEM. In the as-cast samples (Figure 2a), the Fe phase is mainly distributed in the Cu matrix in the form of dendrites with a width of ~4.0 μm and spherical particles with a diameter of ~3.9 μm. When a rolling strain of 1.0 is applied (Figure 2b), the Fe dendrites are tilted and slightly elongated towards the rolling direction, but no significant deformation is observed for the spherical particles. As the rolling strain increases to 3.0 (Figure 2c), the Fe dendrites elongate further and the Fe particles on the dendrites separate from the primary dendrites, whereas the spherical Fe particles develop a tadpole-like shape. With increasing the rolling strain up to 5.0 (Figure 2d), a part of the Fe phase transforms into thin filaments with a thickness of ~0.2 μm, but the other part still exists in the form of tadpole-like particles with long tails.

**Figure 3:**
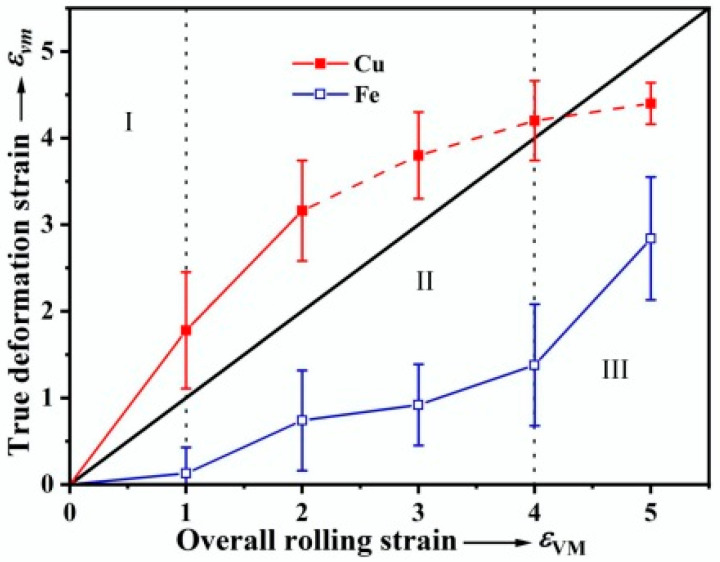
True deformation of the Cu and Fe phases.

To determine the exact deformation degree of each phase, the thickness reduction in the Cu and Fe phases was measured in the longitudinal sections (Figure 1 and Figure 2) and used to calculate the true deformation strain of the two phases ($\varepsilon_{vm}=2/\sqrt{3}\ln\left(d_{0}/d\right)$), as shown in Figure 3. The results indicate that the actual degree of deformation of the Cu phase is much higher than that of the Fe phase. The co-deformation process of the Cu and Fe phases can be divided into three stages: stage I, *ε*_VM_ < 1.0, the Cu matrix accommodates most of the applied strain with no significant deformation of the Fe phase; stage II, 1.0 < *ε*_VM_ < 4.0, the Fe phase starts to deform noticeably, corresponding to a decrease in the plastic strain accommodated by the Cu phase; and stage III, *ε*_VM_ > 4.0, the deformation degree of the Fe phase increases rapidly, while the Cu phase deformation degree reaches saturation. It should be noted that the calculated strain in the Cu matrix is no longer accurate when the overall rolling strain exceeds 2.0, since the Cu matrix starts to undergo dynamic recrystallization which will be discussed later.

### 3.2. Texture Development in Cu and Fe Phases

**Figure 4:**
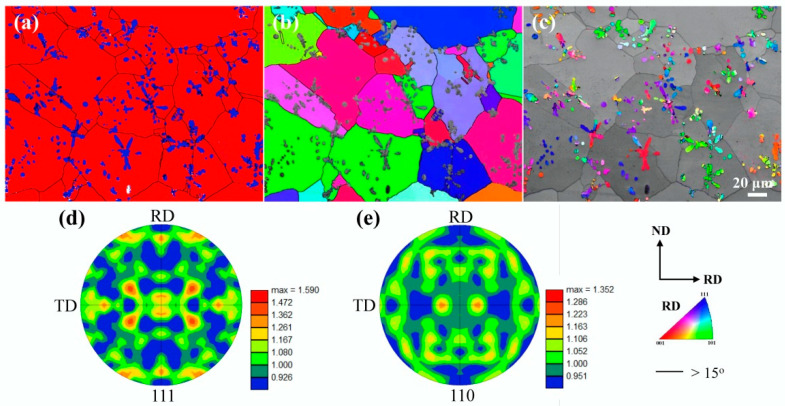
EBSD micrographs of the as-cast Cu-10Fe alloy. (**a**) Phase distribution, (**b**) IPF map of Cu phase, (**c**) IPF map of Fe phase, (**d**) pole figure of Cu phase, and (**e**) pole figure of Fe phase.

Figure 4 shows more microstructural information about the as-cast Cu-10Fe alloy obtained using the EBSD technology. The phase distribution diagram is shown in Figure 4a, where the blue area is the Fe phase, the red area is the Cu matrix, and the black lines are high-angle grain boundaries (HAGB, >15°). Inverse pole figure (IPF) maps of the Cu and Fe phases are shown in Figure 4b and Figure 4c, respectively. The different colors indicate different orientations. It is shown that the orientations are homogeneous inside the Cu grains (Figure 4b) and the spherical Fe particles (Figure 4c), but the Fe dendrites may exhibit different orientations within different trunks (Figure 4c). The pole figures are rather weak for both the Cu phase (Figure 4d) and the Fe phase (Figure 4e), suggesting that they have a nearly random orientation distribution in the as-cast state.

**Figure 5:**
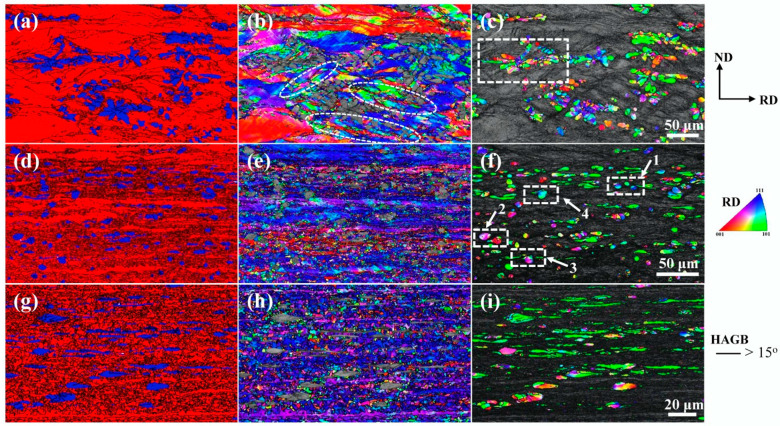
EBSD micrographs of the Cu-10Fe alloy with different cold rolling strains. (**a**–**c**) *ε*_VM_ = 1.0, (**d**–**f**) *ε*_VM_ = 3.0, and (**g**–**i**) *ε*_VM_ = 5.0. (**a**,**d**,**g**) correspond to phase distribution diagrams. (**b**,**e**,**h**) correspond to IPF maps of Cu phase. (**c**,**f**,**i**) correspond to IPF maps of Fe phase.

Figure 5 illustrates typical EBSD maps of the Cu-10Fe alloy after rolling with different strains. In addition to the morphological evolution demonstrated in Figure 1 and Figure 2, these EBSD maps provide more detailed microstructural information. The results show that the shear bands formed at a rolling strain of 1.0 (Figure 5a) have a different orientation from the Cu grains in which they are located (marked by dashed ellipses in Figure 5b). A large number of small equiaxed grains with an average diameter of 1.1 μm are formed in the Cu matrix when the rolling strain reaches or exceeds 3.0 (Figure 5d,g). These small grains usually have a close orientation along the rolling direction (Figure 5e,h), suggesting that they are refined from the Cu lamellar structure. The Fe dendrites at *ε*_VM_ = 1.0 (highlighted by the dotted box in Figure 5c) and the tadpole-like Fe particles at *ε*_VM_ = 3.0 (highlighted by the dotted box in Figure 5f) have a large orientation spread within the grains (corresponding to the color changes), suggesting that these Fe grains have undergone inhomogeneous plastic deformation. Most of the Fe filaments are similar in color at *ε*_VM_ = 5.0 (Figure 5i), indicating that the Fe phase can acquire a stable orientation after undergoing sufficient rolling strains.

**Figure 6:**
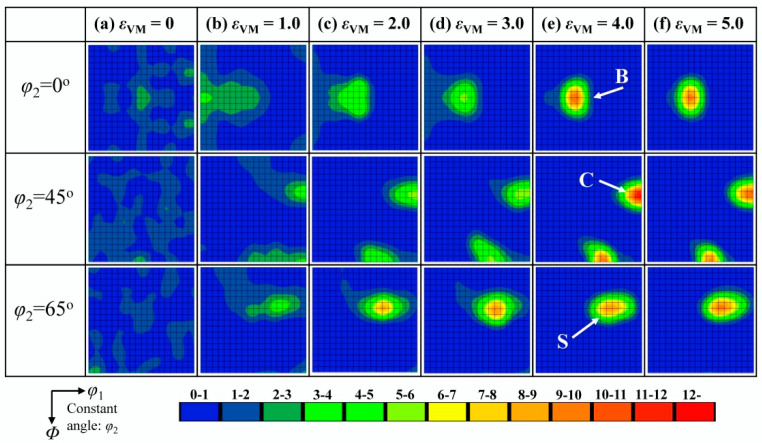
EBSD analysis of the textures in Cu phase at different cold rolling strains. (**a**) *ε*_VM_ = 0, (**b**) *ε*_VM_ = 1.0, (**c**) *ε*_VM_ = 2.0, (**d**) *ε*_VM_ = 3.0, (**e**) *ε*_VM_ = 4.0, and (**f**) *ε*_VM_ = 5.0.

The texture development of the Cu phase during cold rolling, as measured by the large-area EBSD observations, is shown in Figure 6. For the as-cast state, even though the pole figures (Figure 4d) are rather weak and look almost random, a weak intensity of the {011}<322> orientation is present in the three dimensional orientation distribution functions (ODFs) (Figure 6a). As the rolling strain increases from 1.0 to 5.0 (Figure 6b–f), a typical copper-type rolling texture is gradually formed, which develops preferred orientations along the *β*-fiber running from the C orientation {112}<111> through the S orientation {123}<634> to the B orientation {011}<211>.

**Figure 7:**
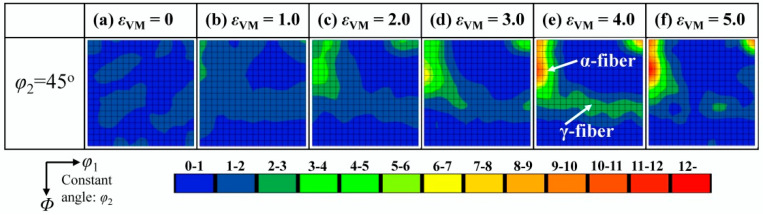
EBSD analysis of the textures in Fe phase at different cold rolling strains. (**a**) *ε*_VM_ = 0, (**b**) *ε*_VM_ = 1.0, (**c**) *ε*_VM_ = 2.0, (**d**) *ε*_VM_ = 3.0, (**e**) *ε*_VM_ = 4.0, and (**f**) *ε*_VM_ = 5.0.

Figure 7 shows the texture development of the Fe phase during cold rolling observed by the large-area EBSD observations. There is no visible texture in the as-cast state (Figure 7a). Upon increasing the rolling strain from 1.0 to 5.0 (Figure 7b–f), typical fiber textures are gradually formed, which are characteristic rolling textures of steel sheets [32]. These fibers are the α-fiber with <110>||RD and the γ-fiber with <111>||ND. It is noted that the γ-fiber has a weaker ODF intensity than the α-fiber; and it disappears at *ε*_VM_ = 5.0.

### 3.3. Microhardness Variations in Cu and Fe Phases

**Figure 8:**
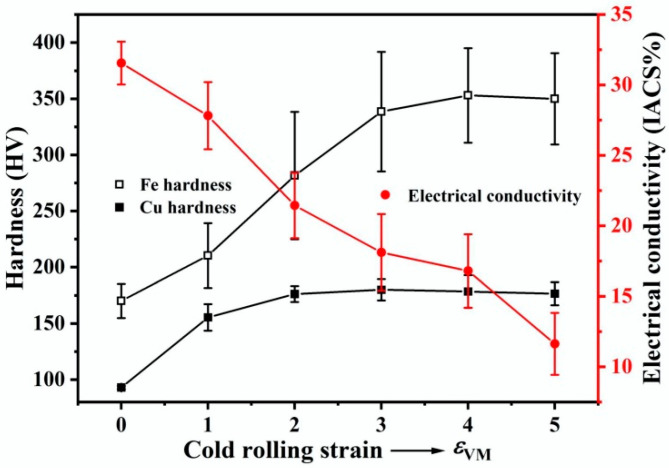
Hardness and electrical conductivity of the Cu-10Fe alloy with different cold rolling strains.

The hardness of the Cu and Fe phases as well as the electrical conductivity of the samples at different cold rolling strains are shown in Figure 8. As the rolling strain increases, the hardness of the Cu phase increases rapidly at first, then rises slightly, and finally gradually stabilizes at ~178 HV. In contrast, the hardness of the Fe phase first rises slowly and then rapidly, before gradually stabilizing at ~350 HV. The electrical conductivity of the samples decreases with increasing rolling strain, from 31.5% IACS in the as-cast state to 11.5% IACS after applying a rolling strain of 5.0.

## 4. Discussion

### 4.1. Co-Deformation Process of Cu and Fe Phases

**Figure 9:**
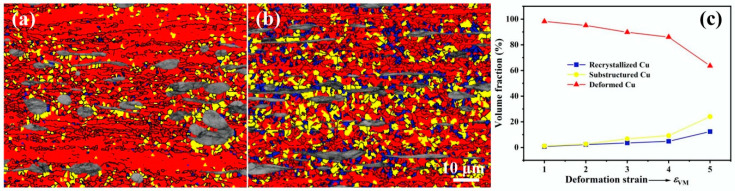
EBSD micrographs of the Cu-10Fe alloy with a rolling strain of (**a**) *ε*_VM_ = 3.0 and (**b**) *ε*_VM_ = 5.0, respectively. The classification was performed using grain orientation spread (GOS) with the following thresholds: recrystallized (blue, GOS < 1°), substructured (yellow, GOS 1–2°), and deformed (red, GOS > 2°) regions. (**c**) Volume fraction of the substructured and recrystallized regions as a function of rolling strain.

The morphological evolution, texture development and microhardness changes in the Cu and Fe phases in the Cu-10Fe alloy during cold rolling have been investigated. The results indicate that the co-deformation process of the Cu and Fe phases is closely dependent on their relative strengths as well as the morphology of the Fe phase. At the initial stage of rolling with *ε*_VM_ < 1.0, since the hardness and deformation resistance of the Cu matrix is much lower than that of the Fe phase (Figure 8), the Cu phase undergoes plastic deformation ahead of the Fe phase [33]. In order to accommodate the more rigid Fe phase, the Cu matrix takes up most of the applied strain (Figure 3), and leads to an inhomogeneous plastic deformation of the Cu matrix characterized by the appearance of shear bands (Figure 1b). When a rolling strain of *ε*_VM_ = 3.0 is applied, the difficult-to-deform Fe phase hinders the plastic flow of the Cu matrix to some extent, which results in enhancing the dislocation density in the Cu matrix near the Fe phase and improving the deformation heat generated between them [12], leading to dynamic recovery or dynamic recrystallization of the Cu matrix around the Fe phase to form some ultrafine grains (Figure 5d). This can be demonstrated more clearly by the EBSD image shown in Figure 9a, where the red, yellow, and blue areas represent the deformed, substructured, and recrystallized microstructures, respectively. As the rolling strain increases to *ε*_VM_ = 5.0, more regions of the Cu matrix undergo dynamic recovery or dynamic recrystallization, as demonstrated in Figure 9b. The volume fraction of the substructured and recrystallized regions as a function of rolling strain is quantified in Figure 9c. The hardness of the Cu matrix saturates at a rolling strain of *ε*_VM_ = 3.0 (Figure 8). This is the result of dynamic recovery, i.e., the dislocation multiplication and annihilation are balanced. The Cu matrix strength decreases slightly when the rolling strain exceeds *ε*_VM_ = 3.0 (Figure 8), and this is attributed to the softening caused by dynamic recrystallization.

**Figure 10:**
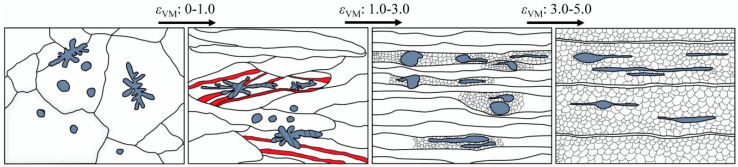
Schematic view of the co-deformation process of the Cu and Fe phases in the Cu-10Fe alloy during cold rolling.

The Fe phase starts to deform plastically when the rolling strain comes to *ε*_VM_ = 1.0 (Figure 3), because at this time the flow stress in the Cu matrix approaches the strength of the Fe phase due to work hardening (Figure 8). However, with increasing strain, the strength of the Fe phase again becomes much higher than that of the Cu phase due to work hardening (Figure 8). This makes further plastic deformation of the Fe phase largely dependent on the surrounding stress concentration caused by the plastic flow of the Cu matrix. When a rolling strain of *ε*_VM_ = 3.0 is applied, the Fe dendrites elongate significantly along the rolling direction, while the spherical Fe particles develop only a tadpole-like shape (Figure 5). The Fe dendrites have complex shapes, and thus the plastic flow of the Cu matrix causes a large stress concentration around them, making them relatively easy to deform plastically [34]. The stress concentration acting on the spherical Fe particles is relatively small due to their high geometrical symmetry, making them unable to elongate as a whole but only shear in the edge regions where the thickness is relatively small, thus forming a tadpole-like shape. The evolution from spherical Fe particles to filaments enhances interfacial load transfer through increased Cu/Fe contact area while promoting strain homogenization [26]. Tadpole-to-filament transition reduces localized Cu matrix straining by enabling coordinated Fe phase deformation [27,28]. With increasing the rolling strain up to *ε*_VM_ = 5.0, the deformation degree of the Fe phase increases significantly (Figure 3) and many Fe filaments are formed (Figure 2d). This is due to the fact that the Fe phases are more likely to deform plastically when the spacing between them becomes smaller [35]. However, a part of the Fe phase is still present in the form of tadpole-like particles, except that the particle tails become longer, suggesting that the spherical shape is not favorable for the formation of Fe fibers. Based on the above discussion, the co-deformation process of the Cu and Fe phases can be summarized in Figure 10.

**Figure 11:**
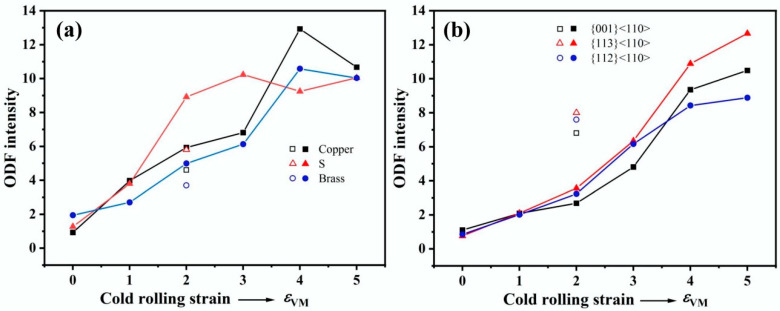
ODF intensities of characteristic texture components of the (**a**) Cu and (**b**) Fe phases in the Cu-10Fe alloy at different cold rolling strains. The hollow symbols indicate the data obtained from pure Cu and pure Fe.

There has been less research aimed at determining the textures developed in two-phase materials compared with single-phase materials. The present study indicates that the Cu and Fe phases develop the same texture components as when they are present alone (Figure 6 and Figure 7). Figure 11a,b display the ODF intensity as a function of rolling strain for the characteristic texture components of the Cu and Fe phases, respectively. For comparison, the ODF intensities of the pure Cu and Fe with a cold rolling strain of *ε*_VM_ = 2.0 are also plotted in Figure 11a and 11b, respectively. It is found that the texture in the Cu phase is enhanced by the presence of the harder Fe phase, and this can be attributed to the extra deformation accommodated by the Cu phase (Figure 3). On the contrary, the texture evolution slows down in the Fe phase. Similar trends have also been observed in the rolled α/β brass [36]. Notably, the decrease in Copper and Brass components at *ε*_vm_ > 4.0 (Figure 11a) is attributed to dynamic recrystallization in the Cu matrix. Dynamic recrystallization generates new grains with random orientations, diluting the rolling texture. This is supported by the increased recrystallized fraction in Figure 9c. Similar phenomena have also been observed in other cold deformed Cu alloys [1,12].

### 4.2. Deformation Mechanism of Fe Phase

**Figure 12:**
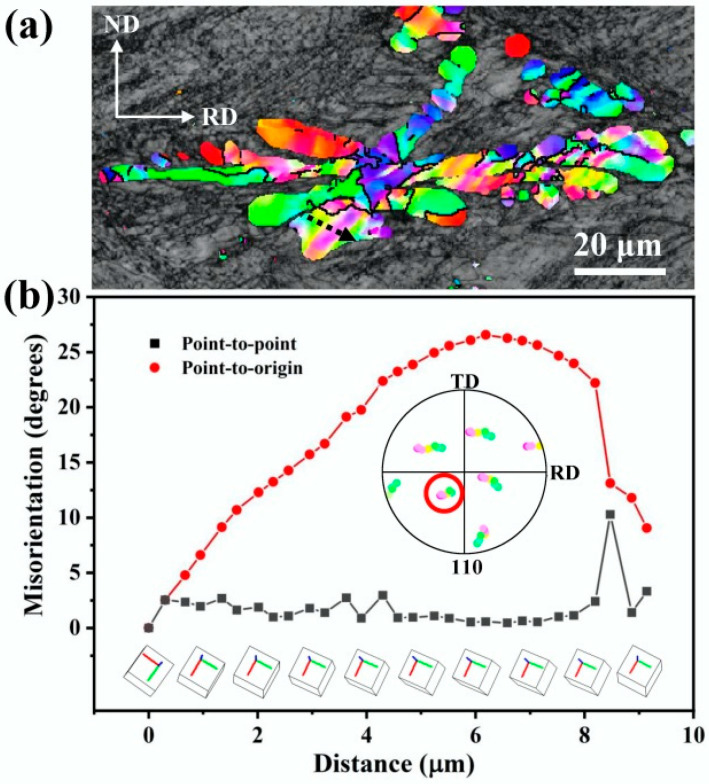
(**a**) Magnified image of the area indicated by the dashed box in Figure 5c. (**b**) Point-to-point and point-to-origin misorientations measured along the arrow direction in (**a**). The inset map in (**b**) is the EBSD pole figure of the local orientations measured along the arrow direction in (**a**).

The local orientation changes in Fe dendrites and spherical Fe particles during cold rolling were examined to explore the deformation mechanism of the Fe phase. Figure 12a is an enlarged image of the boxed area in Figure 5c. It is evident from the figure that after applying a rolling strain of *ε*_VM_ = 1.0, several deformation bands with an angle of 45° to the rolling direction are formed inside the Fe dendrite. A measurement of the local orientations along the arrow direction shown in Figure 12a reveals that a large orientation spread is formed inside the Fe dendrite, which is formed by the continuous slight rotation of the neighboring lattice, as shown in Figure 12b. Plotting these local orientations into a pole figure (inset of Figure 12b) reveals that they share roughly one common (110) pole (indicated by the red circle), which suggests that the Fe matrix rotates around the <110> axis in a slip plane during cold rolling. Taking into account that the slip direction of the bcc crystal is <111>, the {112} plane is considered to be the responsible slip plane in the deformation [33].

**Figure 13:**
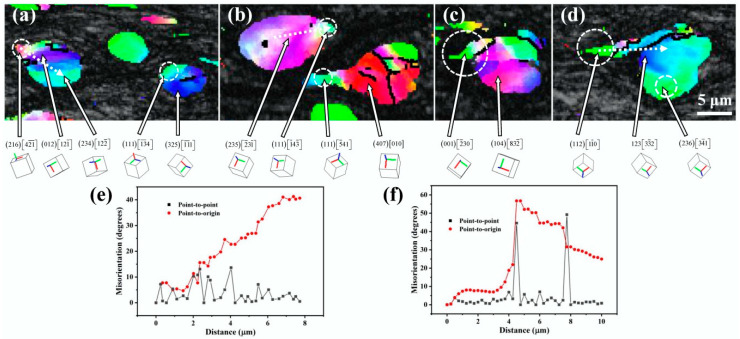
(**a**–**d**) are magnified images of the boxed regions labeled with 1, 2, 3, and 4 in Figure 5f, respectively. (**e**,**f**) illustrate the point-to-point and point-to-origin misorientations measured along the arrows in (**a**,**d**), respectively.

**Figure 14:**
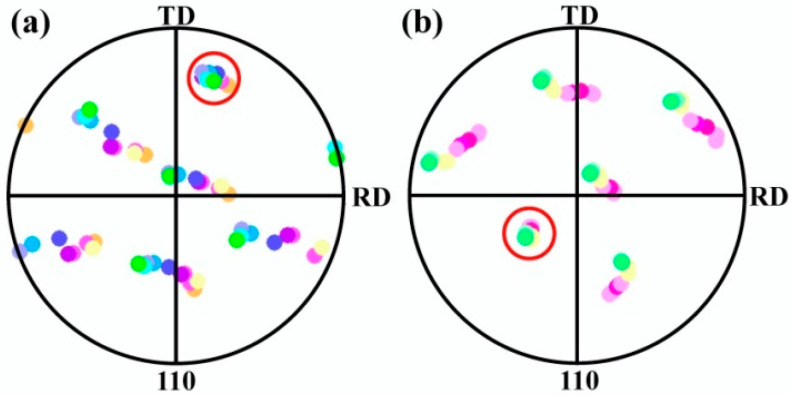
(**a**,**b**) are the EBSD pole figures of the local orientations measured along the arrows in Figure 13a and Figure 13b, respectively.

The areas marked by the dotted boxes labeled with 1–4 in Figure 5f are magnified and shown in Figure 13a–d, respectively. The spherical Fe particles develop into a tadpole-like shape when a rolling strain of *ε*_VM_ = 3.0 is applied. Measurement of the local orientation changes along the arrow direction in Figure 13a reveals that at the initial stage of forming tadpole-shaped particles, a large orientation spread is also formed within the particles, resulting from continuous slight rotations of neighboring lattices, as demonstrated in Figure 13e. By measuring the local orientation changes along the arrow direction in Figure 13d, it is found that as the tadpole-like particles develop, the tails of the particles transform into new grains, and the orientation spread inside the tails becomes relatively homogeneous, as shown in Figure 13f. It is noted that the tails of the tadpole-shaped particles have <142>||RD (Figure 13a) and <143>||RD (Figure 13b) orientations at the beginning of their formation, which gradually shift to <145>||RD (Figure 13b) and <230>||RD (Figure 13c) as the tails become longer, and eventually turn to <110>||RD (Figure 13d) as the tails develop further. The local orientations measured along the arrows in Figure 13a,b are plotted into pole figures as shown in Figure 14a and b. The results show that the local orientations within the tadpole-like Fe particles share one common (110) pole (indicated by the red circles), suggesting that the Fe dendrites and the spherical Fe particles have the same deformation mechanism, i.e., the matrix lattice rotates around the <110> axis in a {112} slip plane during the cold rolling process.

### 4.3. Possible Methods to Promote Fe Fiber Formation

**Figure 15:**
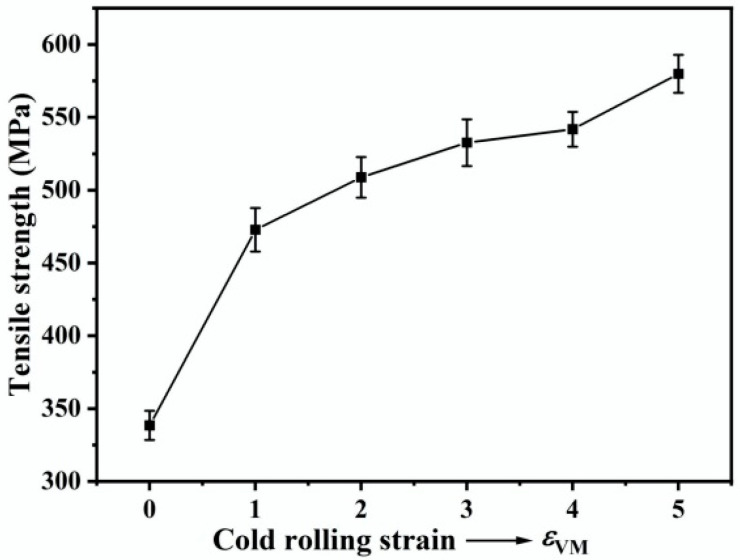
Tensile strength of the Cu-10Fe alloy at different cold rolling strains.

Figure 15 shows the variation in tensile strength of the Cu-10Fe alloy as a function of rolling strain. The strength increases significantly with applying a rolling strain of *ε*_VM_ = 1.0. After that, the strength rises only slightly as the rolling strain increases further to *ε*_VM_ = 4.0. But when the rolling strain is greater than *ε*_VM_ = 4.0, the strength starts to increase rapidly again. The initial rapid increase in strength is attributed to work hardening of the Cu matrix, after which the slight increase in strength is attributed to strength saturation of the Cu matrix and work hardening of the low-volume-fraction Fe phase (Figure 8). Since the hardness of the Cu and Fe phases saturates before the rolling strain reaches *ε*_VM_ = 4.0 (Figure 8), the rapid increase in the tensile strength of the Cu-10Fe alloy after the rolling strain exceeds *ε*_VM_ = 4.0 should be attributed to the fiber strengthening contributed by the formation of a large number of Fe fibers. Clearly, the key to further improving the mechanical properties of the rolled Cu-Fe alloy sheets is to facilitate the formation of Fe fibers.

The results of this work show that the lower strength of the Cu matrix leads to delayed deformation of the Fe phase, and the spherical Fe particles tend to transform into tadpole-shaped particles during rolling. Therefore, increasing the strength of the Cu matrix and avoiding the appearance of spherical Fe particles may be a promising solution to facilitate the formation of Fe fibers. Common methods of strengthening the Cu matrix include solid solution strengthening with the addition of third alloying elements such as Mg [37] and Ag [38], and precipitation strengthening using mechanical-thermal treatments [39]. Additionally, the Cu matrix can also be strengthened by optimizing solidification conditions (e.g., controlling cooling rate) and adjusting composition (such as adding trace elements or modifying Fe content) to refine grain structure and homogenize Fe precipitate distribution [3,7]. The formation of spherical Fe phases in Cu-Fe alloys may be related to the phenomenon of liquid phase separation, which can be hindered by controlling the solidification process [21] and reducing the content of impurities that promote liquid phase separation [40].

## 5. Conclusions

The morphological evolution, texture development, local orientation changes and microhardness variations in the Cu and Fe phases in a Cu-10Fe alloy have been examined under various cold rolling strains, the following results can be drawn:

(1) The as-cast Cu-10Fe alloy consists of equiaxed Cu grains, Fe dendrites and spherical Fe particles. After applying a rolling strain of *ε*_VM_ = 1.0, the equiaxed Cu grains elongate along the rolling direction without significant deformation of the Fe phase. With increasing the rolling strain up to *ε*_VM_ = 3.0, the Cu matrix transforms into a lamellar structure and undergoes dynamic recrystallization in the region close to the Fe phase, while the Fe dendrites elongate along the rolling direction and the spherical Fe particles develop into tadpole-like shapes. When the rolling strain increases to *ε*_VM_ = 5.0, most of the Cu matrix undergoes dynamic recrystallization, with a part of the Fe phase transforming into fibers and the other part remaining as tadpole-like particles with long tails.

(2) During the cold rolling process, the Cu and Fe phases develop the same texture components as when they are present alone but the rate of texture development is somewhat different. Texture development in the Cu phase is enhanced by the presence of the harder Fe phase due to the extra plastic deformation accommodated by the Cu phase. On the contrary, the texture evolution is retarded in the Fe phase.

(3) The tails of tadpole-like particles possess an orientation close to <110>||RD in their initial stages of formation, resulting from the continuous rotation of the matrix lattice around the <110> axis in a {112} slip plane. The tails transform into new grains and achieve a stable orientation of <110>||RD when they develop long enough.

(4) Narrowing the strength gap between the Cu and Fe phases and avoiding the appearance of spherical Fe phase are the keys to facilitating the transformation of the Fe phase into fibers during the rolling process.

## Data Availability

The original contributions presented in this study are included in the article/Supplementary Materials. Further inquiries can be directed to the corresponding author.

## Supplementary Materials

The following supporting information can be downloaded at: https://www.mdpi.com/article/10.3390/ma18112547/s1, Figure S1: A schematic diagram illustrating the key steps of casting, cold rolling, and specimen extraction; Figure S2: Optical micrographs of the Cu-10Fe alloy with different cold rolling strains. (a) ε_VM_ = 2.0, (b) ε_VM_ = 4.0; Figure S3: SEM images of the Cu-10Fe alloy with different cold rolling strains. (a) ε_VM_ = 2.0, (b) ε_VM_ = 4.0; Figure S4: EBSD micrographs of the Cu-10Fe alloy with different cold rolling strains. (a–c) ε_VM_ = 2.0, (d–f) ε_VM_ = 4.0. (a) and (d) correspond to phase distribution diagrams. (b) and (e) correspond to IPF maps of Cu phase. (c) and (f) correspond to IPF maps of Fe phase.
