# Supplementary material for: Co-Deformation Process of Cu and Fe Phases in Cu-10Fe Alloy During Cold Rolling
Source: Materials (Basel). 2025 May 28;18(11):2547. doi: 10.3390/ma18112547 (PMC12156915; doi:10.3390/ma18112547)
Supplement: Supplementary file 1 [file materials-18-02547-s001.zip › materials-3656292-supplementary.pdf]

Supplementary Data

Co-Deformation Process of Cu and Fe Phases in Cu-10Fe Alloy During Cold Rolling

Wei Chen [1,2], Xiaona Hu [1], Jiawei Wang [1,2], Qiuxiang Liu [1], Dan Wu [1,2], Jiang Jiang [1], Qiang Hu [1], Deping Lu [1] and Jin Zou [1,2,*]

[1] Institute of Applied Physics, Jiangxi Academy of Sciences, 7777 Changdong Avenue, Nanchang 330096, China; chenw@alum.imr.ac.cn (W.C.); xnhu13s@163.com (X.H.); jww@whu.edu.cn (J.W.); qx0813@126.com (Q.L.); bird5810761@163.com (D.W.); superjj1981@163.com (J.J.); huqiang1225@163.com (Q.H.); ludeping61@163.com (D.L.)

[2] Jiangxi Key Laboratory of Advanced Copper-Based Materials, 7777 Changdong Avenue,
Nanchang 330096, China

* Correspondence: niatzou@126.com

Supplementary Material Additions:

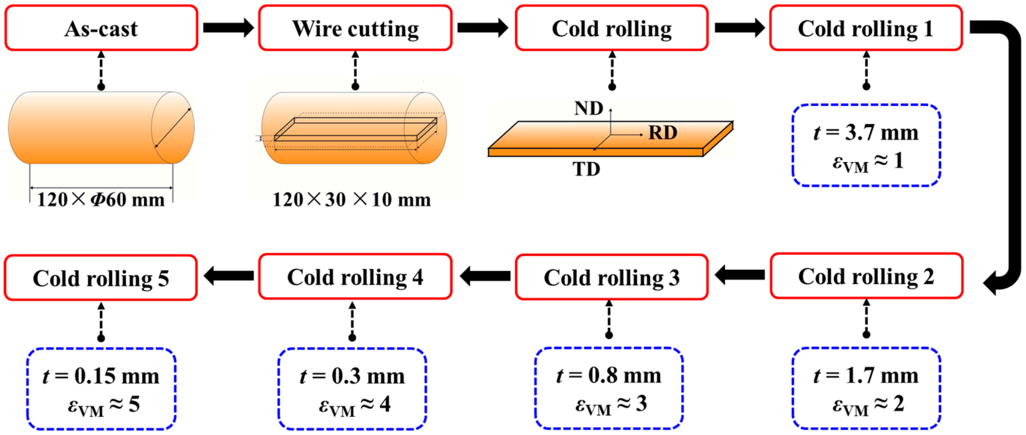

Figure S1. A schematic diagram illustrating the key steps of casting, cold rolling, and specimen extraction.

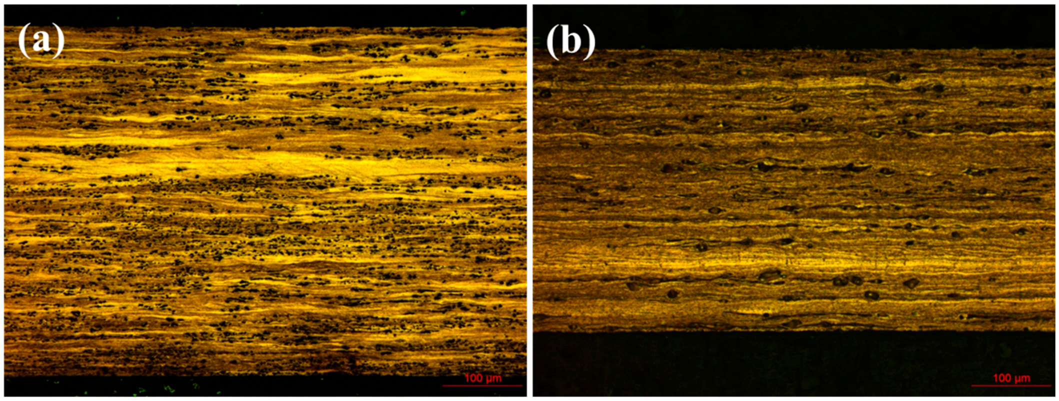

Figure S2. Optical micrographs of the Cu-10Fe alloy with different cold rolling strains. (**a**) $\varepsilon_{VM} = 2.0$, (**b**) $\varepsilon_{VM} = 4.0$.

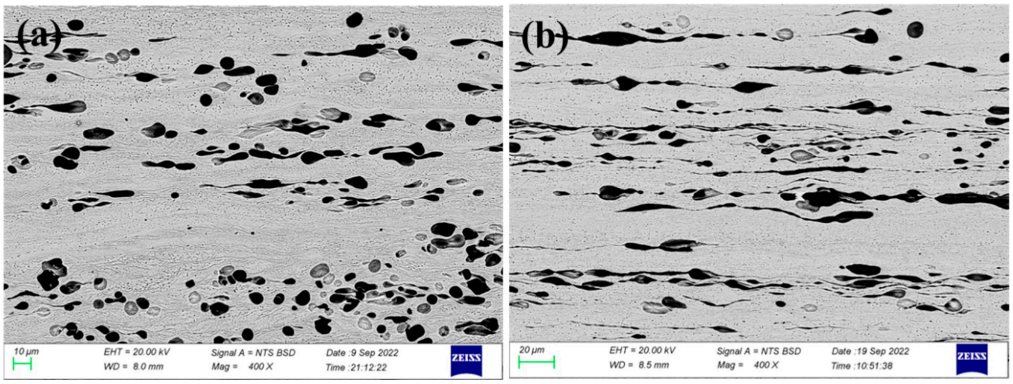

Figure S3. SEM images of the Cu-10Fe alloy with different cold rolling strains. (**a**) $\varepsilon_{VM} = 2.0$, (**b**) $\varepsilon_{VM} = 4.0$.

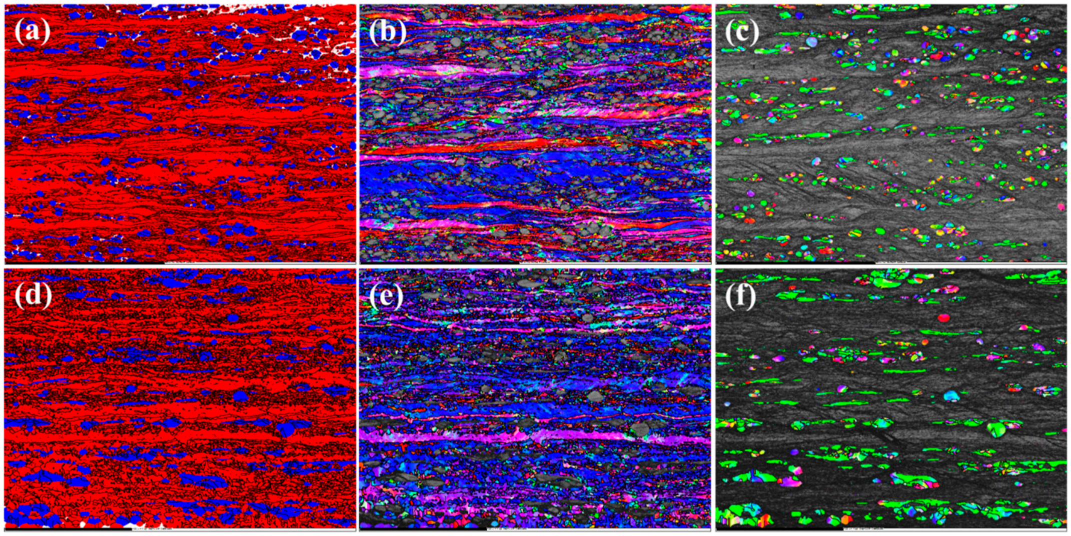

Figure S4. EBSD micrographs of the Cu-10Fe alloy with different cold rolling strains. (**a**–**c**) $\varepsilon_{VM} = 2.0$, (**d**–**f**) $\varepsilon_{VM} = 4.0$. (**a**,**d**) correspond to phase distribution diagrams. (**b**,**e**) correspond to IPF maps of Cu phase. (**c**,**f**) correspond to IPF maps of Fe phase.
